# Cardiovascular disease and diagnosis of advanced prostate cancer

**DOI:** 10.1186/s40959-025-00384-9

**Published:** 2025-10-01

**Authors:** Kevin T. Nead, Allen M. Haas, Jing Zhao, Ting Xiong, Chad Tang, Sharon H. Giordan, Nicholas J. Leeper

**Affiliations:** 1 Department of Epidemiology, University of Texas MD Anderson Cancer Center,, 6900 Fannin Street, Office FHB6.1060, Houston, TX 77030 USA; 2 Department of Breast Radiation Oncology, University of Texas MD Anderson Cancer Center, Houston, TX USA; 3 Department of Health Services Research, University of Texas MD Anderson Cancer Center, Houston, TX USA; 4 Division of Vascular Surgery, Department of Surgery, Stanford University School of Medicine, Stanford, CA USA; 5 Department of Genitourinary Radiation Oncology, University of Texas MD Anderson Cancer Center, Houston, TX USA; 6 Department of Breast Medical Oncology, University of Texas MD Anderson Cancer Center, Houston, TX USA; 7 Stanford Cardiovascular Institute, Stanford, CA USA; 8 Division of Cardiovascular Medicine, Department of Medicine, Stanford University School of Medicine, Stanford, CA USA

## Abstract

**Introduction:**

Cardiovascular disease (CVD) and cancer are the two leading causes of death in the US. Preclinical models support a direct effect of cardiovascular disease (CVD) on accelerated cancer growth and spread. Our objective is to test the hypothesis that individuals with prevalent CVD are at an increased risk of presenting with more advanced prostate cancer at diagnosis.

**Methods:**

We conducted a case–control study in the Surveillance, Epidemiology, and End Results (SEER)-Medicare linked databases from 2010–2019. The analysis was undertaken from October 2024 to February 2025. We included male individuals aged ≥ 67 years diagnosed with invasive prostate cancer with at least two healthcare interactions and evidence of PSA screening in the 3 to 24 months prior to cancer diagnosis. Our exposure of interest was CVD in the 3 to 24 months prior to cancer diagnosis. Our a priori hypothesis tested the odds of prevalent CVD in patients with localized (T1-2 and N0 and M0) versus advanced (T3-4 or N + or M +) prostate cancer at diagnosis.

**Results:**

Our analysis included 12,120 matched individuals, with median age 75 years (interquartile range 71–80), of which 88% were white, 8% were black, and 59% had prevalent CVD. Multivariable adjusted models demonstrated that individuals with advanced prostate cancer at diagnosis had a statistically significant 10% increased odds of prevalent CVD (OR, 1.10; 95% CI, 1.00–1.22; *p* = 0.047). This finding was strongest when examining individuals with regional or distant spread at diagnosis (N + or M + ; OR, 1.19; 95% CI, 1.05–1.34; *p* = 0.006). Further, individuals with Gleason score ≥ 8 disease at diagnosis, had an increased odds of prevalent CVD (OR, 1.07; 95% CI, 1.01–1.13; *p* = 0.020).

**Conclusion:**

We demonstrate an association between prevalent CVD and advanced prostate cancer at diagnosis. Our results may help guide patients regarding personalized screening decisions, given current guidelines recommending shared decision-making, and can be used to facilitate targeted screening approaches.

**Supplementary Information:**

The online version contains supplementary material available at 10.1186/s40959-025-00384-9.

## Introduction

Mechanistic data from multiple preclinical cancer models have demonstrated that cardiovascular disease (CVD) may be causally associated with cancer [1–3]. Induced myocardial infarction (MI) in intestinal adenoma models demonstrates increased tumor burden, potentially secondary to cardiac secreted factors [3]. Similarly, induced MI in models of breast cancer results in increased proliferative index, primary tumor volume, and metastatic spread secondary to MI-induced immunosuppression [2]. We built on these prior studies to demonstrate that individuals with prevalent CVD were more likely to have advanced breast cancer at diagnosis, particularly traditionally indolent receptor subtypes [4]. Here we extend these existing data, and our prior work demonstrating an association between CVD and increased prostate cancer incidence [5], by testing the hypothesis that individuals with prevalent CVD are more likely to have advanced prostate cancer at diagnosis. Our results may have implications for personalized screening decisions. 

## Methods

This case–control study followed Strengthening the Reporting of Observational Studies in Epidemiology guidelines and was exempted from review and informed consent by the University of Texas MD Anderson Cancer Center institutional review board as a secondary analysis of a limited dataset. Human Ethics and Consent to Participate declarations: not applicable.

We utilized Surveillance, Epidemiology, and End Results (SEER)-Medicare to identify men with a first diagnosis of prostate cancer diagnosed from 2010–2019, ≥ 67 years, with ≥ 2 healthcare interactions and PSA screening in the 3–24 months pre-diagnosis, and complete stage and grade information. See eTable 1 for cohort selection. Race and ethnicity were extracted from SEER data obtained from medical records [6]. Race was categorized as Black, White, or other (e.g., American Indian or Alaska Native or Asian or Pacific Islander). Ethnicity was classified as Hispanic or non-Hispanic. Race and ethnicity were included as potential confounding factors. Variable values with unknown or missing data were considered as a separate category or, where necessary to facilitate matching, grouped with other.

We conducted 1:3 greedy nearest neighbor propensity score caliper (0.25) exact matching utilizing factors associated with delayed cancer diagnosis and CVD [4, 7]. The propensity score used factors with evidence for delayed cancer diagnosis: age, race and ethnicity, Medicaid eligibility, urban or rural residence, marital status, region, and quartile of number of health care interactions (unique days with an inpatient or outpatient claims code) in the 3 to 24 months prior to cancer diagnosis [8, 9]. The Gleason score analysis utilized a 1:1 propensity score matched cohort.

We compared prevalent CVD status among individuals with (T3-4 or N + or M +) and without (T1-2 and N0 and M0) advanced prostate cancer at diagnosis. Further, we stratified CVD status according to whether it was or was not derived based on inpatient claims codes, as a proxy for CVD severity. We additionally examined Gleason score at diagnosis comparing those with ≥ 8 versus < 8. We implemented conditional logistic regression adjusting for matching variables and comorbid conditions: chronic obstructive pulmonary disease, diabetes, hypertension, hyperlipidemia, and chronic kidney disease. CVD status and comorbidities were determined using established methods and are outlined in eTable 2. Tests were considered statistically significant if 2-sided P-value < 0.05. Analyses were conducted using SAS (v7.15).

## Results

Our analytic cohort included 12,120 individuals with prostate cancer. Individuals had a median age 75 years (IQR 71–80) including 903 Black (7.5%), 10,659 White (87.9%), and 558 other/unknown race (4.6%) individuals. There were 7,127 (58.8%) individuals with prevalent CVD prior to prostate cancer diagnosis. We identified 9,090 individuals with localized prostate cancer (T1-2 NO MO) matched with 3,030 individuals with advanced prostate cancer (T3-4 or N + or M +). Complete demographic and clinical data are presented in Table 1.

**Table 1:** Demographic data for the primary analytic cohort matched by disease stage at diagnosis (*n* = 12,120)

Characteristic	T1-2 N0 M0 (*n* = 9090)	T3-4 or N + or M + (*n* = 3030)	*P*-Value
**Diagnosis stage**			
T1-2 N0 M0	9090 (100)	NA	NA
T3-4 N0 M0	NA	993 (32.8)	
N + or M +	NA	2037 (67.2)	
**Age at diagnosis, y**			
67–70	2189 (24.1)	735 (24.3)	0.75
71–75	2737 (30.1)	924 (30.5)	
76–80	1943 (21.4)	657 (21.7)	
81–85	1461 (16.1)	455 (15.0)	
≥ 86	760 (8.4)	259 (8.5)	
**Race**			
Black	653 (7.2)	250 (8.3)	0.08
White	8029 (88.3)	2630 (86.8)	
Other/Unknown^a^	408 (4.5)	150 (5.0)	
**Ethnicity**			
Hispanic	515 (5.7)	186 (6.1)	0.33
Non-Hispanic	8575 (94.3)	2844 (93.9)	
**Marital Status**			
Married	6268 (69.0)	2055 (67.8)	0.01
Not Married	2203 (24.2)	775 (25.6)	
Unknown	619 (6.8)	200 (6.6)	
**Rural**			
Urban	7719 (84.9)	2564 (84.6)	0.69
Rural	1371 (15.1)	466 (15.4)	
**Region**			
Northeast	1655 (18.2)	574 (18.9)	0.75
Midwest	1131 (12.4)	386 (12.7)	
Southeast	1973 (21.7)	652 (21.5)	
West	4331 (47.6)	1418 (46.8)	
**Dual Eligibility**			
No	6268 (69.0)	2055 (67.8)	0.33
Yes	2203 (24.2)	775 (25.6)	
**Visits**			
Q1 (1–15)	2488 (27.4)	804 (26.5)	0.74
Q2 (16–27)	2724 (30.0)	912 (30.1)	
Q3 (28–45)	1780 (19.6)	590 (19.5)	
Q4 (46 +)	2098 (23.1)	724 (23.9)	
**Year**			
2010	675 (7.4)	228 (7.5)	0.90
2011	732 (8.1)	247 (8.2)	
2012	728 (8.0)	254 (8.4)	
2013	740 (8.1)	255 (8.4)	
2014	770 (8.5)	240 (7.9)	
2015	986 (10.8)	339 (11.2)	
2016	1125 (12.4)	357 (11.8)	
2017	982 (10.8)	350 (11.6)	
2018	1204 (13.2)	387 (12.8)	
2019	1148 (12.6)	373 (12.3)	
**CKD**	698 (7.7)	297 (9.8)	< 0.01
**COPD**	1067 (11.7)	400 (13.2)	0.03
**Hyperlipidemia**	6918 (76.1)	2221 (73.3)	< 0.01
**Hypertension**	6761 (74.4)	2310 (76.2)	0.04
**Diabetes**	2580 (28.4)	911 (30.1)	0.08
**CVD**	5298 (58.3)	1829 (60.4)	0.04

All data presented as “number (%)”, *P*-values from chi-squared test

*Abbreviations: NA* not applicable, *CKD* chronic kidney disease, *COPD* chronic obstructive pulmonary disease, *CVD* cardiovascular disease

^a^American Indian/Alaska Native, Asian/Pacific Islander

**Fig. 1:**
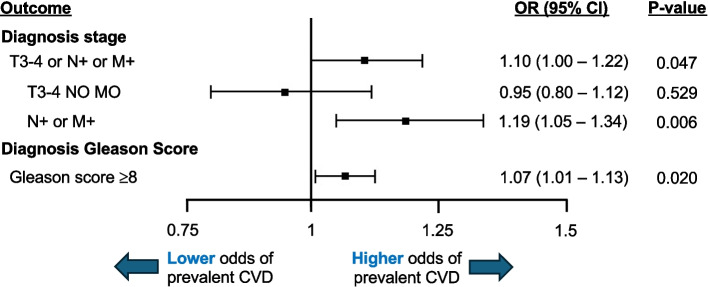
Association of cardiovascular disease with prostate cancer stage and Gleason score, at prostate cancer diagnosis. Forest plot showing the odds ratio (OR) and 95% confidence interval (CI) for the association of prevalent cardiovascular disease (CVD) with prostate cancer stage at diagnosis (reference group: localized prostate cancer [T1-2 and N0 and M0] for all groups) and Gleason score at diagnosis (reference group: Gleason score < 8)

Our primary analysis (Fig. 1) demonstrated that, compared to men with localized prostate cancer, individuals diagnosed with advanced prostate cancer had a statistically significant 10% increased odds of prevalent CVD (1.10; 95% CI, 1.00–1.22; *p* = 0.047). This finding was strongest among individuals with regional or distant prostate cancer at diagnosis (N + or M +; OR, 1.19; 95% CI, 1.05–1.34; *p* = 0.006). More severe CVD was associated with an increased odds of advanced and regional or distant prostate cancer at diagnosis (eTable 3).

We subsequently utilized the same criteria to select individuals with Gleason score ≥ 8 disease at diagnosis 1:1 propensity score matched to individuals with Gleason score < 8 disease at diagnosis (see eTable 4 for baseline and demographic characteristics). We found that individuals with Gleason score ≥ 8 disease at diagnosis, versus < 8, had a 7% increased odds of prevalent CVD (OR, 1.07; 95% CI, 1.01–1.13; *p* = 0.020; Fig. 1).

## Discussion

In this case–control study we found that men with prevalent CVD prior to prostate cancer diagnosis have a greater risk of presenting with advanced prostate cancer, specifically regional and distant spread or high Gleason score (≥ 8) disease, compared to those without prevalent CVD. Additionally, our results suggest that more severe CVD may be associated with a greater risk of advanced prostate cancer at diagnosis. Importantly, our analyses were conducted among a PSA screened cohort, with frequent healthcare interactions, propensity score matching on factors associated with delayed cancer diagnosis, and adjusting for extensive demographic factors and comorbid conditions. While our observed effect sizes were modest, our results have significant potential public health implications given the high population level prevalence of CVD. Our results are consistent with prior data from our group showing that CVD is associated with more advanced breast cancer at diagnosis [4] and increased prostate cancer incidence [5]. Importantly, the associations observed in our population level analyses are directly supported by evidence of a causal effect of CVD on cancer incidence and severity from multiple preclinical cancer models [1–3].

A growing body of literature supports a causal relationship between CVD and cancer. In preclinical models, heart failure following MI results in enhanced intestinal tumor growth [3], early hypertrophic cardiac remodeling promotes tumor growth and metastases via direct cardiovascular and cancer crosstalk [1], and MI-induced immune reprogramming results in an immunosuppressive state that contributes to the growth and spread of breast cancer [2]. Further, multiple large-scale analyses of real-world data accounting for shared risk factors, including smoking, have found that individuals with CVD, particularly atherosclerotic CVD, are at an increased risk of cancer [5, 10]. Our investigations of cancer stage and grade at diagnosis extend prior population level analyses and directly test hypotheses generated from preclinical work and real-world datasets.

Our data in prostate cancer are consistent with our analysis showing that CVD is associated with more advanced breast cancer at diagnosis among traditionally indolent subtypes [4]. Specifically, we previously found an association between prevalent CVD and risk of locally advanced or metastatic breast cancer at diagnosis (OR, 1.10; 95% CI 1.03–1.17). Interestingly, stratification of this analysis by breast cancer receptor subtype demonstrated that the observed association was limited to the most traditionally indolent subtype (hormone receptor–positive and ERBB2-negative; OR, 1.12; 95% CI 1.04–1.21). This observation was consistent with preclinical data where CVD induced accelerated breast tumor cell growth and spread was demonstrated in hormone receptor–positive models of breast cancer [2, 11, 12]. This led us to hypothesize that more traditionally indolent cancer types, such as prostate cancer, may have a longer time window to be effected by CVD prior to initial diagnosis. Our analysis and findings in prostate cancer presented here further support this hypothesis.

Our study has limitations. Our analysis utilized a retrospective study design that does not prove causality and is susceptible to residual bias and confounding. We were unable to control for some potential confounding factors, including smoking status. Our cohort was primarily white and only included individuals 67 years of age and older, which may impact the generalizability of our findings. Further we were unable to determine duration of CVD, account for risk factors known to be associated with more aggressive disease, including family history, or examine prostate cancer histologic subtypes due to limited power.

In conclusion, we demonstrate an association between CVD and advanced prostate cancer at diagnosis. As current guidelines recommend a shared decision-making model regarding prostate cancer screening [13], our results provide important additional data that may inform individual decisions and facilitate targeted screening approaches. 

## Supplementary Information

Supplementary Material 1

## Data Availability

The datasets used to conduct this study are available upon approval of a research protocol from the National Cancer Institute. Instructions for obtaining these data are available at: https://healthcaredelivery.cancer.gov/seermedicare/obtain/.
