# Supplementary material for: Cardiovascular disease and diagnosis of advanced prostate cancer
Source: Cardiooncology. 2025 Oct 1;11:85. doi: 10.1186/s40959-025-00384-9 (PMC12486819; doi:10.1186/s40959-025-00384-9)
Supplement: Supplementary file 1 — Supplementary Material 1 [file 40959_2025_384_MOESM1_ESM.docx]

**Online-Only Supplement**

eTables

**eTable 1**: Analytic cohort selection.

| Criteria | N | Percent of Previous Step |
| --- | --- | --- |
| 1. Select patients with primary prostate cancer between 2010 and 2019. | 534,833 | - |
| 2. Select patients who are age 67 and older. | 323,009 | 60.4% |
| 3. Select patients with continuous part A and B coverage and no HMO coverage for the year prior to diagnosis. | 167,026 | 51.7% |
| 4. Select patients with complete staging data and without in situ disease. | 96,376 | 57.7% |
| 5. Remove patients with unclear CVD status in the 3-24 months before diagnosis. [Any CVD vs. No CVD] | 84,244 | 87.4% |
| 6. Remove patients who did not have a PSA test in the 3-24 months before diagnosis. | 60,859 | 72.2% |
| 7. Remove patients who did not have at least two healthcare visits in the 3-24 months before diagnosis. | 60,746 | 99.8% |
| 8. Remove patients without grade data at diagnosis. | 58,196 | 95.8% |

CVD, cardiovascular disease

**eTable 2**: Approach to identify exposures, outcomes, and covariables.

| **Variables** | **Codes/Algorithms** |
| --- | --- |
| Prostate Cancer | ICD-O-3: C619 |
| PSA | CPT/HCPCS: 84152, 84153, G0103 |
| Chronic obstructive pulmonary disease (derived from CMS Chronic Conditions Data Warehouse) | ICD9: 490, 496, 491.0, 491.1, 491.8, 491.9, 492.0, 492.8, 494.0, 494.1, 491.20, 491.21, 491.22 ICD10: J40, J42, J41.0, J41.1, J41.8, J43.0, J43.1, J43.2, J43.8, J43.9, J44.0, J44.1, J44.9, J47.0, J47.1, J47.9 |
| Cardiovascular disease, hyperlipidemia, hypertension, chronic kidney disease, diabetes | Defined per: Bell CF, Lei X, Haas A, et al. Risk of Cancer After Diagnosis of Cardiovascular Disease. JACC CardioOncol. Aug 2023;5(4):431-440. doi:10.1016/j.jaccao.2023.01.010   - Cardiovascular disease status was determined from the 24-months prior to cancer diagnosis using 1) 2 or more separate International Classification of Disease (ICD), 9^th^ (ICD-9) or 10^th^ (ICD-10) codes from an outpatient setting at least 30 days apart, 2) one inpatient code, or 3) one procedure code.   - Individuals with only one outpatient cardiovascular disease code were excluded due to unclear cardiovascular disease status. - Comorbidities were identified by the presence of any relevant code in the two years prior to diagnosis |

**eTable 3**: Association of CVD diagnosed by inpatient or outpatient claims codes with odds of prostate cancer stage and grade at diagnosis.

| Model | OR (95% CI) |
| --- | --- |
| **Advanced** (T3-4 or N+ or M+)^#^ |  |
| No CVD | Reference^ |
| Outpatient CVD | 1.08 (0.98 – 1.20) |
| Inpatient CVD | 1.37 (1.15 – 1.64) |
|  |  |
| Inpatient vs. Outpatient CVD* | 1.27 (1.08 – 1.49) |
| **Locally Advanced** (T3-4 NO MO)^#^ |  |
| No CVD | Reference |
| Outpatient CVD | 0.95 (0.80 – 1.12) |
| Inpatient CVD | 0.87 (0.60 – 1.26) |
|  |  |
| Inpatient vs. Outpatient CVD | 0.92 (0.65 – 1.30) |
| **Regional or Distant** (N+ or M+)^#^ |  |
| No CVD | Reference |
| Outpatient CVD | 1.16 (1.02 – 1.31) |
| Inpatient CVD | 1.59 (1.29 – 1.95) |
|  |  |
| Inpatient vs. Outpatient CVD | 1.37 (1.14 – 1.65) |
| **Gleason Score** (≥ 8 vs <8 disease) |  |
| No CVD | Reference |
| Outpatient CVD | 1.06 (1.00 – 1.12) |
| Inpatient CVD | 1.16 (1.05 – 1.28) |
|  |  |
| Inpatient vs. Outpatient CVD | 1.10 (1.00 – 1.20) |
| CI, confidence interval; OR, odds ratio  ^#^Versus T1-2 and N0 and M0; *Reference group is Outpatient CVD | |

**eTable 3**: Demographic data for the secondary analytic cohort matched by Gleason score at diagnosis (n=29088).

| **Characteristic** | **Gleason score <8 (n = 14544)** | **Gleason score ≥8 (n = 14544)** | **P-Value** |
| --- | --- | --- | --- |
| **Age at diagnosis, y** |  |  |  |
| 67-70 | 3270 (22.5) | 3341 (23.0) | <0.01 |
| 71-75 | 4810 (33.1) | 4769 (32.8) |  |
| 76-80 | 3518 (24.2) | 3429 (23.6) |  |
| 81-85 | 2215 (15.2) | 2058 (14.2) |  |
| 86+ | 731 (5.0) | 947 (6.5) |  |
| **Race** |  |  |  |
| Black | 1148 (7.9) | 1178 (8.1) | 0.75 |
| White | 12395 (85.2) | 12350 (84.9) |  |
| Other/Unknown^a^ | 1001 (6.9) | 1016 (7.0) |  |
| **Ethnicity** |  |  |  |
| Hispanic | 776 (5.3) | 794 (5.5) | 0.64 |
| Non-Hispanic | 13768 (94.7) | 13750 (94.5) |  |
| **Marital Status** |  |  |  |
| Married | 10004 (68.8) | 9878 (67.9) | 0.27 |
| Not Married | 2914 (20.0) | 2983 (20.5) |  |
| Unknown | 1626 (11.2) | 1683 (11.6) |  |
| **Dual Eligibility** |  |  |  |
| No | 13328 (91.6) | 13225 (90.9) | 0.03 |
| Yes | 1216 (8.4) | 1319 (9.1) |  |
| **Rural** |  |  |  |
| Urban | 12249 (84.2) | 12183 (83.8) | 0.29 |
| Rural | 2295 (15.8) | 2361 (16.2) |  |
| **Region** |  |  |  |
| Northeast | 2971 (20.4) | 2991 (20.6) | 0.82 |
| Midwest | 1610 (11.1) | 1656 (11.4) |  |
| Southeast | 3573 (24.6) | 3544 (24.4) |  |
| West | 6390 (43.9) | 6353 (43.7) |  |
| **Visits** |  |  |  |
| Q1 (1-15) | 3474 (23.9) | 3483 (23.9) | 0.97 |
| Q2 (16-27) | 4647 (32.0) | 4613 (31.7) |  |
| Q3 (28-45) | 2901 (19.9) | 2928 (20.1) |  |
| Q4 (46+) | 3522 (24.2) | 3520 (24.2) |  |
| **Year** |  |  |  |
| 2010 | 1444 (9.9) | 1400 (9.6) | 0.93 |
| 2011 | 1430 (9.8) | 1434 (9.9) |  |
| 2012 | 1212 (8.3) | 1210 (8.3) |  |
| 2013 | 1193 (8.2) | 1197 (8.2) |  |
| 2014 | 1229 (8.5) | 1264 (8.7) |  |
| 2015 | 1406 (9.7) | 1429 (9.8) |  |
| 2016 | 1482 (10.2) | 1512 (10.4) |  |
| 2017 | 1665 (11.4) | 1644 (11.3) |  |
| 2018 | 1693 (11.6) | 1734 (11.9) |  |
| 2019 | 1790 (12.3) | 1720 (11.8) |  |

All data presented as “number (%)”; P-values from chi-squared test

Abbreviations: NA, not applicable

^a^American Indian/Alaska Native, Asian/Pacific Islander
